# 

**Published:** 1891-05-02

**Authors:** 


PRICE ONE PENNY.

No. 240, Vol. X.— May 2nd, 1891.

[Registered at the General Post Office as a Newspaper for transmission in the United Kingdom and Abroad.]

WITH EXTRA NURSING SUPPLEMENT.

Per Annum, 6s. 6d.; post free.

Monthly Parts, 6d.; post free 8d.

Ditto per Annum, 8s., post free.

### THE HOSPITAL

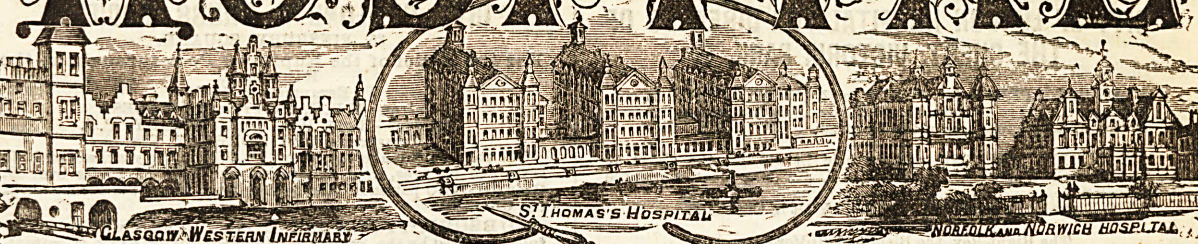

A WEEKLY JOURNAL OF

Science, Medicine, Nursing and Philanthropy.

SATURDAY, MAY 2nd, 1891.

A Funeral Oration . . . . . . . . 49

Passing Topics.—An Early Victim . . . . 50

The Doctor's Armchair.—"Died of Acute Pneumonia" . 51

The Microscope in Medicine—By Frank J. Wethered, M.D., XIII.—Examination of the Blood . . . 52

The Radcliffe Infirmary, Oxford . . . . 53

Turning Over New Leaves.—The Medical Annual . 53

Notes and News . . . . . . . 54

Annotations.—Rational Dress—The Royal Free asks for £20,000—Gifts and Gratitude . . . . 56

Editor's Letter Box.—Is Philanthropy a Duty?—Prescriptions and their Owners . . . . . 60

Scraps and Gleanings . . . . . . 55

The Practitioner's Mirror and Retrospect:—

Medicine.—Anæmia . . . . . . 57

Surgery.—Ingleby Lectures: Lecture IV. . . 58

Therapeutics.—Piperazidine . . . . 59

New Drugs Appliances, and Things Medical.—Coca-Wine—Pure Cane Sugar . . . . . 59

The Royal British Nurses' Association and the Nurse Training Schools . . . . . 60

The Lords' Committee . . . . . . 60

The Student of Medicine.—The Students' Bookshelf—Appointments—Vacancies—Scholarships and Prizes.

EXTRA SUPPLEMENT—THE NURSING MIRROR.

En Passant.—Short Items—Figures for Nurses to Note and Remember—Torquay Institute—Service for the King—Yesterday and To-day—Holidays . . . . xxv
Lectures on Surgical Ward Work and Nursing.—By Alex. Miles, M.B. Edin., C.M., F.R.C.S.E.—Lecture XXII.—Scott's Dressing . . . . . xxvi
Miss Nightingale and the Registration of Nurses xxvi
Nursing Medals and Certificates.—St. Bartholomew's Hospital . . . . . . . . xxvii
For Reading to the Sick.—Life's Education . . xxvii
Notes from Australia . . . . . . xxviii
Everybody's Opinion.—Pensions for Servants—Male Nurses—The Private Nurse . . . . . xxix
Presentations . . . . . . . xxix
Notes and Queries . . . . . . xxix
The Invalid Children's Aid Association.—Part I.—What the Association Does . . . . . xxx
Where to Go.—Amusements and Relaxation . . xxx

